# Sporadic Dup15q Syndrome Presenting With Developmental Delay, Intellectual Disability, Attention-Deficit/Hyperactivity Disorder, and Epilepsy: A Case Report

**DOI:** 10.7759/cureus.87458

**Published:** 2025-07-07

**Authors:** Josef Finsterer, Awini Barwari

**Affiliations:** 1 Neurology, Neurology and Neurophysiology Center, Vienna, AUT

**Keywords:** adhd, atomoxetine, chromosome-15 microduplication, developmental delay, epilepsy, intellectual disability

## Abstract

Chromosome 15q duplication (Dup15q) syndrome is a rare genetic disorder that presents with a range of psychiatric and neurological symptoms. To date, no cases have been reported involving a patient with a 300 kb microduplication on chromosome 15 presenting with developmental delay, intellectual disability, attention-deficit/hyperactivity disorder (ADHD), and epilepsy. We describe a 20-year-old male diagnosed with Dup15q syndrome at the age of nine. His early development was marked by congenital strabismus, global developmental delay, and intellectual disability, which required enrollment in a special education program until age 11. He also exhibited hyperactivity, aggression, and self-injurious behavior. Since then, both his motor and cognitive functions have progressively declined. At age 14, he was diagnosed with ADHD. Treatment with atomoxetine, methylphenidate, and risperidone provided partial symptom relief. However, escitalopram triggered episodes of severe tantrums and was subsequently discontinued. From the age of 16, he began experiencing epilepsy, characterized by focal seizures, generalized tonic-clonic seizures, and absence seizures. Valproic acid (VPA) was effective in significantly reducing his seizure activity. This case highlights that Dup15q syndrome associated with a 300 kb microduplication can predominantly affect the central nervous system and may respond favorably to atomoxetine, methylphenidate, risperidone, and VPA. Dup15q syndrome should be considered in the differential diagnosis of individuals presenting with developmental delay, intellectual disability, ADHD, and epilepsy.

## Introduction

Chromosome 15q duplication (Dup15q) syndrome is a rare neurodevelopmental disorder clinically characterized by intellectual disability, impaired motor coordination, autism spectrum disorder (ASD), hypotonia, and intractable epilepsy [1]. The duplications may be inherited (approximately 15% of cases) or occur de novo (approximately 85%) [1]. The additional genetic material typically originates from a maternal isodicentric 15q11.2-q13.1 supernumerary chromosome - idic(15) - which contains two extra copies of 15q11.2-q13.1, resulting in tetrasomy for this region (approximately 60-80% of cases). Alternatively, a maternal interstitial duplication of 15q11.2-q13.1 may occur, resulting in trisomy for this region (approximately 20-40% of cases). Individuals with a maternal isodicentric 15q11.2-q13.1 chromosome generally exhibit more severe phenotypes than those with an interstitial duplication [1].

In some cases, at least one additional copy of genetic material is found within the Prader-Willi/Angelman Critical Region of the affected 15q11.2-q13.1 chromosome [2]. The UBE3A gene, which encodes ubiquitin ligase E3A, is believed to play a central role in the development of Dup15q syndrome phenotypes, although other genes - such as *SNHG14*, *HERC2*, *FOXO1*, *EHPB2*, and *RORA* - may also contribute to phenotypic variability [3,4].

The estimated prevalence of Dup15q syndrome ranges from 1 in 30,000 to 1 in 60,000 children worldwide [2]. Clinically, the syndrome is primarily associated with psychiatric manifestations [5], including schizophrenia and schizoaffective disorders [5,6], ASD [5,7], intellectual disability [8], behavioral issues [8], sleep disturbances [9], and neurological symptoms such as epilepsy - ranging from complex partial seizures and tonic-clonic seizures (TCS) to tonic seizures, atypical absences, Lennox-Gastaut syndrome, infantile spasms, myoclonic absences, and electrical status epilepticus - and hypotonia [7,10-12].

Developmental delay, behavioral problems, speech delay, dysmorphic facial features, and seizures may be observed in both maternal and paternal duplications. However, ASD is more frequently associated with maternal duplications, whereas paternal duplications are typically linked to asymptomatic or very mildly affected individuals [10].

To date, no case has been reported of a patient with Dup15q syndrome presenting with congenital strabismus, developmental delay, intellectual disability, attention-deficit/hyperactivity disorder (ADHD), and epilepsy associated with a 300 kb Dup15q (chromosomal coordinates: 22784523-3085096).

## Case presentation

**Figure 1:**
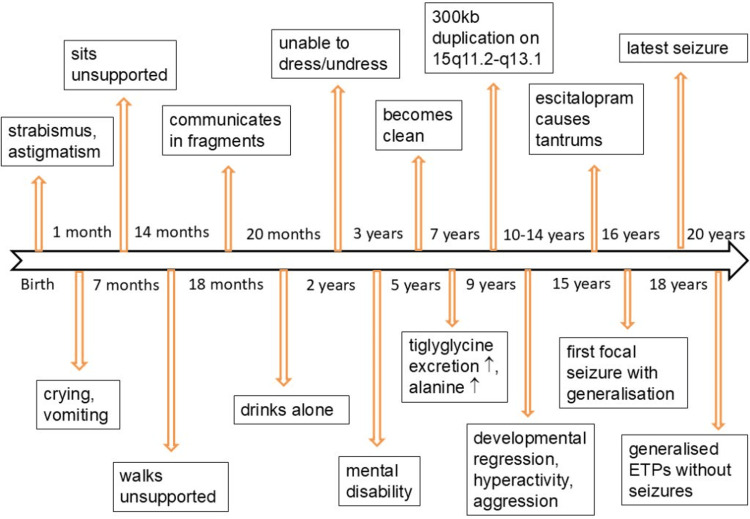
Timeline of the disease course in the index patient ETP, epilepsy typical potentials

The patient was a 20-year-old man, 163 cm tall and weighing 51.5 kg, who was born by caesarean section to unrelated parents. He developed normally during the first few weeks of life. Thereafter, he presented with frequent crying, strabismus, astigmatism, and recurrent vomiting (Figure 1). At seven months of age, he was able to sit without support, and at 14 months, he began walking independently. By 18 months, he spoke his first words (“mama,” “papa,” and “water”) and could communicate using two- to three-word sentences. At 20 months, he was able to drink independently. However, by the age of two years, he remained unable to dress or undress himself. At the age of three, he was diagnosed with a mental disability. He achieved continence at the age of five.

Screening at the age of seven revealed slightly elevated tiglyglycine excretion in the organic acid profile and mildly increased alanine levels in the amino acid panel. Cerebral MRI findings were reported as normal. Ophthalmologic examination showed hyperopia, astigmatism, and strabismus divergens. At nine years of age, comparative genomic hybridization identified a 300 kb duplication on chromosome 15q11.2-q13.1, spanning from breakpoint 1 (position 22784523) to breakpoint 2 (position 3085096). This duplication was de novo, as it was not detected in either parent. Cytogenetic analysis revealed a normal karyotype, and multiplex ligation-dependent probe amplification results were also normal. Although he attended a special school until the age of 11, he did not learn to read or write.

In the subsequent years, he experienced regression of both mental and motor abilities, accompanied by significant behavioral issues, including extreme hyperactivity, aggressive outbursts, compulsive behaviors, self-injurious tendencies, clothing biting, stereotypies, and symptoms consistent with ADHD. He was started on risperidone at the age of 14. Due to limited effectiveness, the dose was increased, and atomoxetine was added. Escitalopram was introduced at age 15. From the age of 16, he developed tantrums occurring 10-15 times daily, each accompanied by fatigue and urinary incontinence. These episodes were attributed to escitalopram, and the medication was discontinued.

Also, at age 16, he underwent surgical repair for an inguinal hernia. That same year, he began experiencing focal seizures with secondary generalization (Figure 1). The seizures responded well to valproic acid (VPA), and his last TCS occurred at the age of 18.

**Figure 2:**
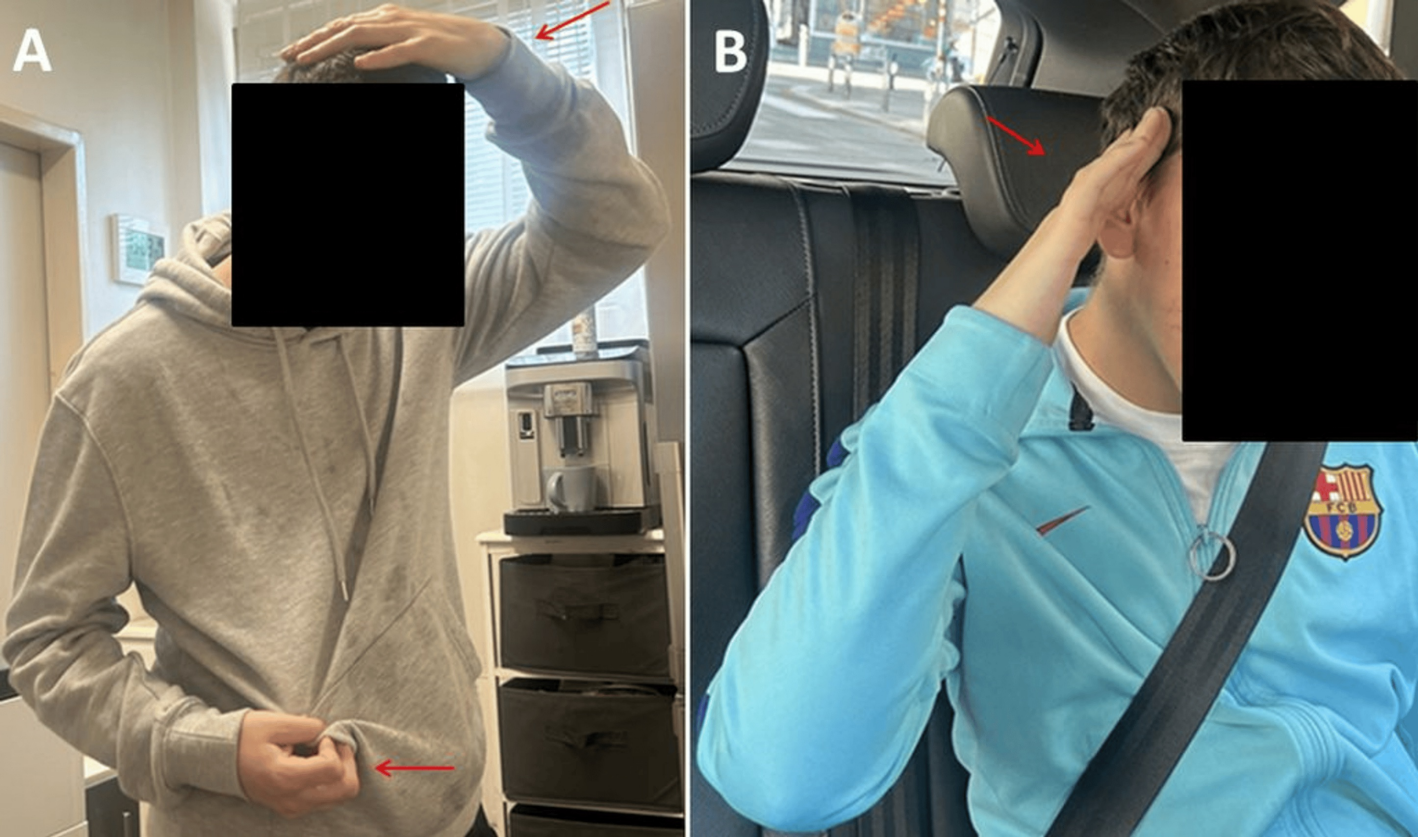
Stereotypic hand movements and positions observed in the patient (A and B)

At the age of 20, a clinical neurological examination revealed strabismus divergens, hypermetropia, a persistently open mouth, and an inability to follow prompts. The patient exhibited constant movement, an inability to sit or lie still for extended periods, recurrent myoclonus, stenciling behavior (placing the left hand into the mouth), and an implied stenciling posture (Figure 2). Additional findings included reduced pain sensitivity, absent tendon reflexes, and frequent backward reclining of the upper body while walking. A notable café-au-lait spot extended from the left shoulder blade to the left upper extremity.

**Figure 3:**
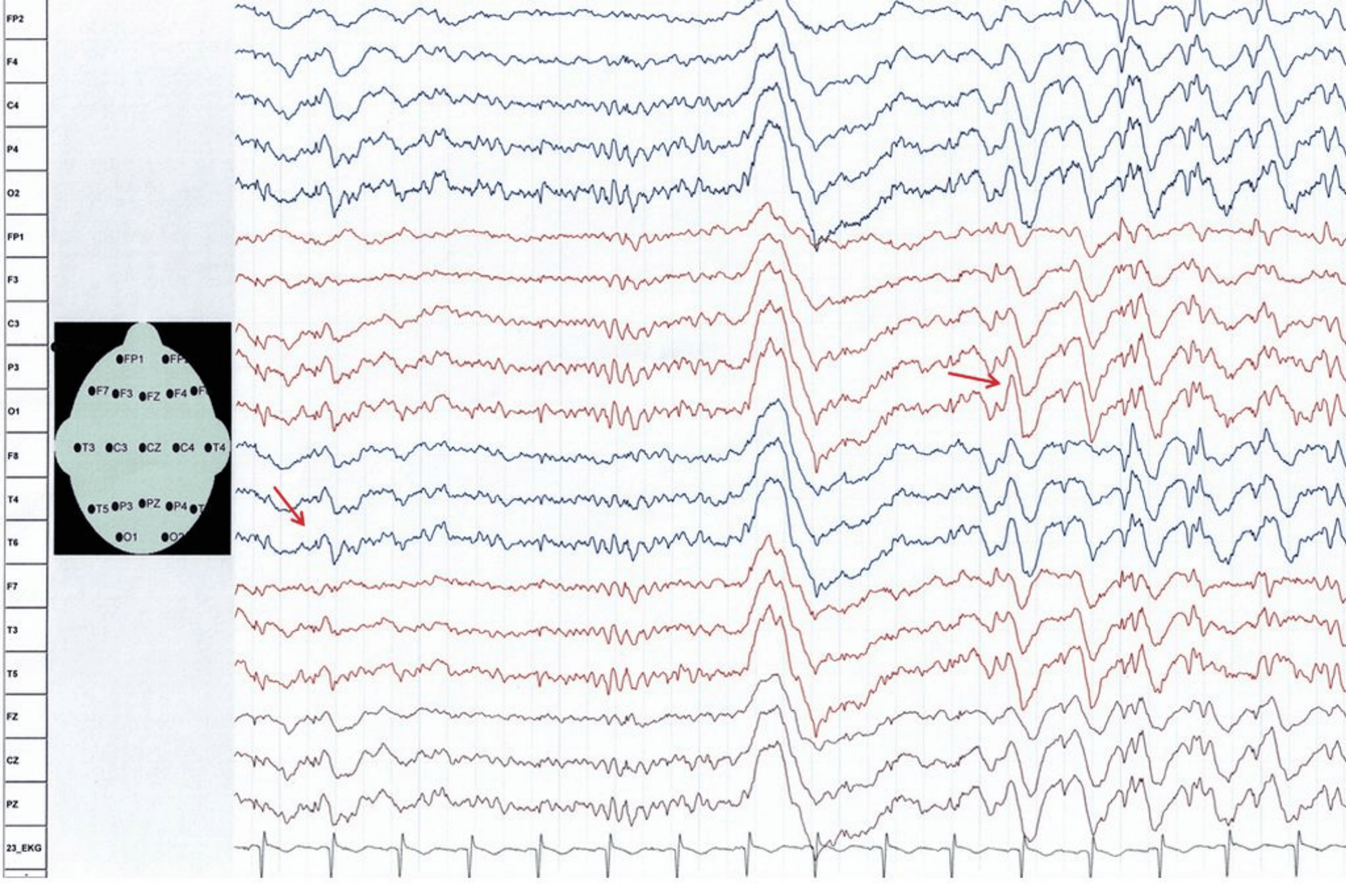
Routine scalp EEG recordings (reference montage) at age 20 showing recurrent episodes of generalized sharp waves and spike-wave complexes

Blood tests at age 20 were largely unremarkable except for a vitamin D deficiency. EEG revealed background slowing at 7-8 Hz, with activity spreading to the frontal regions and showing appropriate reactivity to eye opening and closing. Additionally, recurrent episodes of generalized sharp waves and spike-wave complexes were recorded (Figure 3). Brain MRI performed at age 21 was normal, consistent with previous imaging findings. 

The patient is currently receiving risperidone (7.5 mg/day), atomoxetine (100 mg/day), methylphenidate (37 mg/day), and VPA (2,500 mg/day). While receiving this treatment, he continues to live with his parents and attends a center for people with disabilities between 9:00 a.m. and 3:00 p.m. Afterward, he stays at home and is almost constantly active, but is still unable to feed himself, bathe, dress and undress, or use a mobile phone. His attention span was limited; he could watch television for no more than 30 minutes and displayed emotional responses to specific scenes. Sleep patterns were normal. He received pediatric care until the age of 19, after which he transitioned to a neurological care facility for adults.

## Discussion

The patient presented here is of particular interest for several reasons. First, the patient carried a 300 kb microduplication on chromosome 15q11.2-q13.1, a variant that has not been previously reported. Although similar duplications have been identified in asymptomatic individuals, likely due to lower penetrance [13], the role of this microduplication in the patient’s clinical presentation remains uncertain. However, a causal relationship is supported by the absence of alternative diagnoses and the fact that both 15q11.2-q13.1 microduplications [3-8] and microdeletions have been associated with severe clinical manifestations [14,15]. The most well-known deletion syndromes involving this region are Angelman syndrome and Prader-Willi syndrome (PWS) [14,15].

Angelman syndrome is caused by a 15q11.2-q13.1 deletion in approximately two-thirds of cases. It is characterized by severe intellectual disability, absent speech, frequent bouts of laughter accompanied by hand-flapping, microcephaly, macrostomia, hypoplastic maxilla, a puppet-like gait, ataxia, epileptic seizures with triphasic delta activity (maximal over the frontal regions), a cheerful disposition, hyperactivity without aggression, short attention span, easy excitability, sleep disturbances with reduced sleep need, increased heat sensitivity, coarsening of facial features, and thoracic scoliosis [14].

PWS is clinically marked by significantly reduced fetal movements, abnormal fetal positioning, pronounced neonatal hypotonia, failure to thrive in infancy, followed by hyperphagia leading to obesity, along with physical features such as a furrowed forehead, downturned mouth, almond-shaped blue eyes, strabismus, blond hair, short stature, small hands and feet, distally narrowed fingers, scoliosis, hypogonadism, and hypogenitalism. Developmental anomalies, intellectual disability, and behavioral disturbances are also typical [15].

While the index patient shared some features with Angelman syndrome, including limited speech, reduced attention span, mild excitability, hyperactivity, and seizures, only a few phenotypic features of PWS were present, namely strabismus, intellectual disability, and behavioral issues. Importantly, Dup15q syndrome typically does not manifest as either Angelman syndrome or PWS.

Second, the patient exhibited several classic features of Dup15q syndrome, including cognitive impairment, behavioral disturbances, epilepsy, congenital strabismus, reduced pain perception, hypoacusis, speech difficulties, and mild genital anomalies (phimosis). However, some typical features of Dup15q syndrome were absent, such as scoliosis, cryptorchidism, hyperphagia, excessive weight gain, eczema, early puberty, recurrent respiratory infections, low forehead, palpebral fissures, flat nasal bridge, snub nose, forward-flaring nostrils, long philtrum, micrognathia, high-arched palate, low-set ears, full lips, and a flat occiput [16]. Additionally, the patient presented with atypical features not previously associated with Dup15q syndrome, including café-au-lait spots, astigmatism, inguinal hernia, and frequent temper tantrums. The absence of tendon reflexes suggested a possible neuropathy or myopathy; however, the patient was unable to tolerate nerve conduction studies or needle electromyography. While neuropathy has not been reported in Dup15q syndrome, rhabdomyolysis and myopathy have been documented [16].

Third, the therapeutic approach - combining atomoxetine, methylphenidate, and risperidone - led to significant improvement in ADHD and ASD symptoms and cessation of temper tantrums, albeit with some side effects. Atomoxetine is commonly associated with decreased appetite, headache, drowsiness, abdominal discomfort, nausea, vomiting, elevated blood pressure, and tachycardia. Other possible adverse effects include irritability, mood swings, insomnia, anxiety, depression, tics, dizziness, mydriasis, constipation, indigestion, rash, itching, fatigue, and weight loss [17]. Methylphenidate may cause dizziness, headache, hypertension, appetite suppression, diarrhea, abdominal pain, psychomotor agitation, anxiety, blurred vision, chest discomfort, tachycardia, nausea, nervousness, priapism, insomnia, aggression, chills, confusion, dark-colored urine, slurred speech, and weight loss [18]. While it remains speculative which symptoms were due to Dup15q syndrome and which were medication-related, a temporary reduction in atomoxetine and methylphenidate doses led to a worsening of symptoms. Given the known association of Dup15q syndrome with sudden unexpected death in epilepsy [19] and gastrointestinal disorders [20], particular caution is warranted regarding the cardiac and gastrointestinal side effects of these medications.

## Conclusions

This case highlights that Dup15q syndrome primarily affects the central nervous system, presenting with developmental delay, intellectual disability, ADHD, and epilepsy. In this patient, the most effective treatments for ADHD were atomoxetine, methylphenidate, and risperidone. Epileptic symptoms responded well to VPA. Dup15q syndrome should be considered in the differential diagnosis of individuals with developmental delay, intellectual disability, ADHD, and epilepsy. Genetic testing using microarray analysis is recommended in patients presenting with developmental regression, intellectual disability, ADHD, and epilepsy to avoid overlooking underlying microdeletion or microduplication syndromes.
